# A longitudinal study of chiropractic use among older adults in the United States

**DOI:** 10.1186/1746-1340-18-34

**Published:** 2010-12-21

**Authors:** Paula Weigel, Jason M Hockenberry, Suzanne E Bentler, Maksym Obrizan, Brian Kaskie, Michael P Jones, Robert L Ohsfeldt, Gary E Rosenthal, Robert B Wallace, Fredric D Wolinsky

**Affiliations:** 1 Department of Health Management and Policy, College of Public Health, the University of Iowa, Iowa City, Iowa, USA; 2 Department of Internal Medicine, Carver College of Medicine, the University of Iowa, Iowa City, Iowa, USA; 3 Center for Research on the Implementation of Innovative Strategies into Practice, Iowa City Veterans Administration Medical Center, Iowa City, Iowa, USA; 4 Department of Biostatistics, College of Public Health, the University of Iowa, Iowa City, Iowa; 5 Kyiv School of Economics, and Kyiv Economics Institute, Kyiv, Ukraine; 6 Department of Health Management and Policy, School of Rural Public Health, Texas A&M University Health Science Center, College Station, Texas, USA; 7 Department of Epidemiology, College of Public Health, the University of Iowa, Iowa City, Iowa, USA

## Abstract

**Background:**

Longitudinal patterns of chiropractic use in the United States, particularly among Medicare beneficiaries, are not well documented. Using a nationally representative sample of older Medicare beneficiaries we describe the use of chiropractic over fifteen years, and classify chiropractic users by annual visit volume. We assess the characteristics that are associated with chiropractic use versus nonuse, as well as between different levels of use.

**Methods:**

We analyzed data from two linked sources: the baseline (1993-1994) interview responses of 5,510 self-respondents in the Survey on Assets and Health Dynamics Among the Oldest Old (AHEAD), and their Medicare claims from 1993 to 2007. Binomial logistic regression was used to identify factors associated with chiropractic use versus nonuse, and conditional upon use, to identify factors associated with high volume relative to lower volume use.

**Results:**

There were 806 users of chiropractic in the AHEAD sample yielding a full period prevalence for 1993-2007 of 14.6%. Average annual prevalence between 1993 and 2007 was 4.8% with a range from 4.1% to 5.4%. Approximately 42% of the users consumed chiropractic services only in a single calendar year while 38% used chiropractic in three or more calendar years. Chiropractic users were more likely to be women, white, overweight, have pain, have multiple comorbid conditions, better self-rated health, access to transportation, higher physician utilization levels, live in the Midwest, and live in an area with fewer physicians per capita. Among chiropractic users, 16% had at least one year in which they exceeded Medicare's "soft cap" of 12 visits per calendar year. These over-the-cap users were more likely to have arthritis and mobility limitations, but were less likely to have a high school education. Additionally, these over-the-cap individuals accounted for 58% of total chiropractic claim volume. High volume users saw chiropractors the most among all types of providers, even more than family practice and internal medicine combined.

**Conclusion:**

There is substantial heterogeneity in the patterns of use of chiropractic services among older adults. In spite of the variability of use patterns, however, there are not many characteristics that distinguish high volume users from lower volume users. While high volume users accounted for a significant portion of claims, the enforcement of a hard cap on annual visits by Medicare would not significantly decrease overall claim volume. Further research to understand the factors causing high volume chiropractic utilization among older Americans is warranted to discern between patterns of "need" and patterns of "health maintenance".

## Background

Complementary and Alternative Medicine (CAM) use in the United States has been examined over the past twenty years, however the patterns of use have not been consistently described due to the heterogeneity of underlying study methods [1-10]. The Institute of Medicine's (IOM) 2005 Report on CAM suggested additional studies were needed to better understand all CAM therapies being used by the American public, the populations that use them, and what is known about how those services are provided [11]. The IOM suggestion was in response to the high rate of growth in both the utilization of and expenditures for CAM over the prior decade.

Among the various CAM modalities, chiropractic is the most identifiable, and one of the largest and most established in the United States [12]. Regulated in all fifty states and the District of Columbia, it is commonly covered by both private and public health insurance plans, with coverage mandated in 46 states [13]. As evidence of its therapeutic validity has grown in the treatment of lower back pain, so has the demand for chiropractic services.

Prior to the 2005 IOM Report there were six national surveys of CAM use in the United States that identified chiropractic as a separate CAM category. Annual chiropractic prevalence rates employing these survey data vary from 6.8%-7.6% [3-5] to 10.1%-11% [1,2] and as high as 16% [6]. While these estimates were for use across the 1990's, the variance in reported rates raises questions about the methods used to ascertain individuals' levels of chiropractic use. More recently, the 2007 National Health Interview Study (NHIS) reported an annual prevalence of "chiropractic or osteopathic manipulation" of 8.6% [7]. Davis and colleagues examined the Medical Expenditure Panel Survey (MEPS) data, which included claims data to validate self-reported use of health services, and estimated 12.6 million adult users of chiropractic in 2006 [8], which based on the U.S. Census [14] results in a prevalence rate of 5.6%.

The wide range of annual prevalence figures for chiropractic use in the United States reflects variability in study design, survey definitions and categories, time periods, and populations. Seven of the studies [1-7] relied solely on self-reported use. As such, reported prevalence figures may be inaccurate representations of actual use among U.S. adults. And while the seven national surveys provide annual U.S. chiropractic prevalence figures, they are single-year estimates that range non-contiguously over the 1990 to 2007 period.

Beyond differences in study design, this body of research provides little insight into health, demographic, and socioeconomic factors associated with chiropractic use. Understanding who uses chiropractic and why are salient research questions posed by the IOM Report [11]. While these seven surveys measure chiropractic use among all adults over the age of 18 (and more recently include the category of use among children under 18 years of age [7]), none have specifically concentrated on chiropractic use, longitudinal patterns of use, or the characteristics of users.

Furthermore, none of these studies have provided insight into the use of chiropractic by older adults [9]. It is important to understand the use of chiropractic in this age group because of both clinical differences in these patients due to age-related frailty, as well as the fact that Medicare uses public funds to pay for chiropractic for its beneficiaries. Wolinsky and colleagues were the first to address the use of chiropractic services by this cohort in the early nineties, and to include a longitudinal element (i.e., a four-year observation period) to the research [9]. We contribute to this literature by examining longitudinal patterns of chiropractic use over 15 years as well as the factors associated with use versus nonuse. The 15-year period allows us to observe variability in chiropractic utilization across calendar years and to explore the stability of annual prevalence over time.

A second contribution of this paper is that we examine characteristics associated with two types of chiropractic users--those that exceed Medicare's "soft cap" of 12 visits in any given calendar year, and those that use fewer chiropractic services. While Medicare does not have a "hard cap" on annual chiropractic visits, it has stipulated that more than 12 visits in any calendar year would likely not be medically necessary and hence may not be coverable [15]. An estimate by the Office of Inspector General (OIG) put the amount spent by Medicare in 2001 on medically unnecessary chiropractic services at more than $251 million, or 55% of total chiropractic services provided to Medicare beneficiaries [15]. Thus, our analyses add a new dimension to understanding utilization variability within an older population uniformly covered by Medicare.

## Methods

### Data

Our analyses were performed using two linked data sources: (1) the baseline (1993-1994) interview responses of self-respondents in the Survey on Assets and Health Dynamics Among the Oldest Old (AHEAD); and, (2) the Medicare carrier claims for those respondents from 1993 to 2007. The design and sampling approach in the AHEAD have been well described elsewhere [16-19]. All analyses are weighted to adjust for the over-sampling of African-Americans, Hispanics, and residents of Florida.

### Sample

There were 7,447 older adults who completed baseline AHEAD interviews in 1993-1994. A total of 1,937 people were excluded from the analytic sample due to (a) the inability to link their Medicare claims (N = 802), (b) being in a managed care Medicare plan at baseline (N = 605), or (c) not being a self-respondent at baseline (N = 530). The final analytic sample consisted of 5,510 individuals, some of whom were censored post-baseline due to death (N = 3,369) or subsequent enrollment in managed care (N = 988). In previous work propensity score re-weighting was used to address the potential sample selection bias introduced by these exclusion criteria; however, such adjustments did not meaningfully alter the results and thus were not used here [20].

### Measuring Chiropractic Use

Chiropractic visits were identified by using the Health Care and Financing Administration's (HCFA) specialty provider code for chiropractors in the Medicare carrier claims file. Claims were aggregated to the individual level in each calendar year of service, as well as across the entire period for which the user was in the sample. Users were partitioned into two groups: those exceeding the 12 chiropractic visit "soft-cap" in any calendar year (high volume users) and those with 12 or fewer annual chiropractic visits in any calendar year (lower volume users).

### Framework for selection of covariates

The AHEAD survey data contains a litany of information on individuals, the totality of which has not been available in previous studies of chiropractic use. In order to bring structure to our inclusion of covariates, we selected them based on Andersen's Behavioral Model of Health Services Use [21]. This model highlights predisposing characteristics that play a role in predicting and explaining health services use in general, and for our analysis, chiropractic use. Variability in demographic factors, such as age, gender, and race could be expected to play a role in explaining chiropractic utilization variation. Social structure variables like education, marital status, and income might also influence why services are sought. Personal enabling resources like having a job, having supplemental insurance, and being able to drive a car would predictably improve access to chiropractic. Need for services can be differentiated by "evaluated" need, such as that identified by a health provider (i.e. arthiritis), or "perceived" need, such as how people view their own health status, functional limitations, psycho-social state, or experience symptoms of pain and illness. Health behavior and lifestyle choices, such as smoking, alcohol consumption, and weight also arguably reveal individual preferences for health that may affect demand for chiropractic. Prior health services utilization measures indicate individual propensity to use health services and prior access to these services. Measures of physician supply, rural-urban characteristics, and distance to a chiropractic college are also included in our model to provide indicators of access and possible familiarity with chiropractic as a health profession. Geographic location measures may reflect differences in regional preferences for chiropractic. All covariates were obtained from baseline interview responses to the AHEAD survey, with the exception of the distance to chiropractic college measure, which was calculated as the distance between a subject's baseline census tract and that of the nearest chiropractic college.

### Demographic, socioeconomic and geographic variables

Demographic covariates are age at baseline, sex, race, and marital status. Socioeconomic measures included educational attainment, income distribution (quintiles), the number of supplemental health insurance policies (zero vs. one or more), whether the respondent was working for pay at baseline, and whether the subject was able to drive a car or not. We included a set of indicators measuring geographic location based on the Health Resources and Services Administration's (HRSA) ten region definition [22], with the Midwest region as the reference group. A measure of rurality was also included, defined by whether a person lived in a non-metropolitan (rural) or metropolitan (non-rural) area. The final measure of geographic interest was distance to nearest chiropractic college. This was included because relative nearness to a chiropractic college might influence local demand for chiropractic care, and chiropractic college graduates may be more likely to locate closer to these institutions thereby increasing supply of the service. Distance was re-coded into two categorical levels: near (under 150 miles to the nearest chiropractic college) and far (greater than 150 miles to the nearest chiropractic college).

### Health and health services use measures

Disease history and comorbidity were measured by participants' responses to survey questions about whether they were ever told by a medical doctor they had a specific health condition. The health conditions included were arthritis, cancer, any heart condition, diabetes, lung disease, hip fracture, or hypertension. In order to reflect the extent of a respondent's comorbidity, we re-coded the count of comorbid conditions into four categories: zero, one (reference category), two, or three or more comorbid conditions. Self-rated health measures at baseline assessed each respondent's view of their own health in terms of "excellent", "very good", "good", "fair", or "poor".

Functional health status was measured in multiple ways. The first was how the respondent answered the question "Are you often bothered by pain?"(yes/no). In addition to the standard activities of daily living (ADLs) and instrumental activities of daily living (IADLs), the AHEAD respondents were asked about five additional measures of upper and lower body limitations to further assess physical impairment. These measures were 'difficulty picking up a dime', 'difficulty lifting ten pounds', 'difficulty pushing or pulling large objects', 'difficulty climbing a flight of stairs', and 'difficulty walking several blocks'. We included pain, ADLs, IADLs, and the additional measures of physical function in our analysis because these are conditions likely to be associated with seeking chiropractic care.

Because pain and lack of physical function are also associated with depression and cognition in older adults, we also included measures of depressive symptoms based on a respondent's score on the Centers for Epidemiologic Studies Depression (CES-D) [23] and measures of cognition based on their scores from the Telephone Interview for Cognitive Status (TICS-7) [24]. In assessing depressive symptoms we classified individuals into three categories: zero depressive symptoms (reference group), one or two depressive symptoms, or three or more depressive symptoms. Similarly we classified cognitive function into three discrete groups: zero-to-ten on the TICS-7 (low cognitive functioning), eleven-to-thirteen on the TICS-7 (normal functioning, and the reference category), and fourteen-to-fifteen on the TICS-7 (high cognitive functioning).

Health lifestyle related factors included cigarette smoking, alcohol consumption, and body mass. Cigarette smoking and alcohol consumption could be related to a person's way of coping with physical pain [25]. Respondents were asked to describe themselves as a current smoker, former smoker, or someone who has never smoked. We re-coded these responses into a single indicator of 'never smoked' versus 'current and/or former smoker' in order to discern between those who might have other underlying health conditions from smoking from those who never smoked. Regarding alcohol consumption, respondents were asked if they ever drank beer, wine or liquor, and if they did how many drinks they averaged per week. In this analysis we used the 'ever drink' variable indicating that a respondent has at least one alcoholic drink during a week vs. never drinking alcohol. BMI measures (kg/m^2^) were included to reflect a potential association between carrying excess weight and back pain. In our study we have four BMI categories: obese ≥ 30 BMI, overweight 25 ≤ BMI < 30, normal weight 18.5 ≤ BMI < 25, and underweight < 18.5 BMI. The normal weight group was the reference category.

Two measures of health services use in the twelve months prior to baseline interview were included: hospital stays (raw count) and the number of physician visits. With regard to the measure of physician visits, the AHEAD survey asks how many times the respondent talked to a medical doctor about their health in the last 12 months, so this measure does not capture visits to non-MD health providers. These are included as baseline indicators of access to health services as well as health status markers. To capture non-linearities in outpatient services use this variable was re-coded into four levels based on the number of physician visits: one or fewer physician visits, two to three physician visits, four to six physician visits, and seven or more physician visits. The reference category was two to three physician visits. A third variable was included to represent the supply of physicians at the local level, as measured by the number of active, nonfederal MDs per 1,000 people in the respondent's county of residence.

### Analytic Approach

Two separate binomial logistic regression was used to identify factors associated with (1) chiropractic use, and (2) conditional upon any use, to identify factors associated with high levels of annual chiropractic use (i.e., those exceeding a "soft cap" of 12 visits in a calendar year versus lower levels of annual use). The logistic regression models used forced entry that included all covariates described above to determine the odds of using chiropractic versus not using. The odds ratios for each of the covariates in the second regression are of being in the high user group versus not being in the high user group. No interaction terms were hypothesized or included in these analyses. In each logistic regression we followed standard procedures for model development and evaluation [26-28].

## Results

### Descriptive

Among the 5,510 subjects there were 15,716 primary and secondary chiropractic visits in the 15-year period. There were 806 users resulting in a period prevalence of 14.6%. The mean annual prevalence of chiropractic use was 4.8% (range 4.1% - 5.4%) and the average number of visits per chiropractic user was 19.5. To test statistical significance we used t-tests for continuous variables and χ2 tests for indicator variables.

Group means for all variables across nonusers and users appear in Table 1. Based on a comparison of the means of the sub-samples, chiropractic users were on average younger than nonusers, a higher percentage were white, had more education and higher income, had more insurance policies, were able to drive, and were married at baseline. Geographically, a lower percentage of chiropractic users lived in the Northeast, Mid-Atlantic, Southwest, and Mountain regions while a higher percentage lived in the North-Central, Midwest, and Pacific-Northwest regions of the United States. A higher percentage of chiropractic users lived in rural areas.

**Table 1:** Baseline means of demographic, socioeconomic, and geographic variables for nonusers and users of chiropractic

	Entire Analytic Sample (N = 5,510)	Nonusers (N = 4,704)	Users (N = 806)	p-value
**Demographic Factors**				
Age	77.4 (5.76)	77.6 (5.87)	76.0 (4.87)	< .0001
Male	38.1%	37.8%	39.7%	0.319
Race				
White	84.8%	83.1%	94.8%	< .0001
African American	10.2%	11.5%	2.4%	< .0001
Hispanic	3.9%	4.2%	2.7%	.0413
Other	1.1%	1.2%	0.2%	< .0064
Marital Status				
Married	50.3%	48.2%	62.8%	< .0001
Never Married	3.4%	3.6%	2.0%	0.0192
Separated/Divorced	5.0%	5.2%	3.7%	0.0697
Widowed	41.3%	43.0%	31.5%	< .0001
**Socioeconomic Factors**				
Educational Attainment				
Grade School Only	25.5%	26.6%	19.0%	< .0001
Some High School	17.2%	17.5%	15.2%	0.0981
High School	30.4%	29.5%	35.6%	0.0005
Some College	26.9%	26.4%	30.2%	0.0238
Income Distribution				
Income - First Quintile	15.6%	16.9%	8.0%	< .0001
Income - Second Quintile	29.9%	30.9%	24.0%	< .0001
Income - Third Quintile	13.0%	13.2%	12.4%	0.5572
Income - Fourth Quintile	18.7%	17.9%	23.3%	0.0003
Income - Fifth Quintile	22.7%	21.0%	32.2%	< .0001
One or More Health Insurance Policy	74.9%	73.5%	82.5%	< .0001
Working for Pay	8.8%	8.4%	11.3%	0.0007
Able to Drive a Car	67.9%	64.8%	85.6%	< .0001
**Geographic Location Measures**				
Northeast	6.1%	6.4%	4.5%	0.0442
New York/New Jersey	12.7%	12.7%	12.7%	0.9471
Mid-Atlantic	9.1%	9.5%	6.6%	0.0081
Southeast	17.2%	17.5%	15.2%	0.1035
North-Central	20.6%	19.8%	25.2%	0.0005
Mid-West	4.8%	4.3%	8.0%	< .0001
South-West	10.8%	11.5%	7.1%	.0002
Mountain	3.2%	3.4%	2.0%	0.0354
West	11.4%	11.2%	12.9%	0.1539
Pacific-Northwest	3.7%	3.4%	5.8%	0.0009
Rural	24.8%	24.3%	27.5%	0.0493
Distance to Chiropractic College - Far	62.6%	62.7%	62.4%	0.8651
**Disease History and Comorbidity**				
No Comorbid Conditions	22.7%	22.0%	26.4%	0.0062
One Comorbid Condition	32.5%	32.2%	34.1%	0.3026
Two Comorbid Conditions	25.1%	25.5%	22.6%	0.0801
Three or More Comorbid Conditions	19.7%	20.3%	16.9%	0.029
Arthritis Ever	24.7%	24.5%	25.8%	0.4134
Cancer Ever	12.9%	13.0%	12.4%	0.6119
Heart Condition Ever	28.9%	29.6%	25.0%	0.0086
Diabetes Now	12.4%	12.8%	10.3%	0.0466
Lung Disease Ever	9.4%	9.9%	6.3%	0.0009
Psych Problems Ever	7.0%	6.9%	7.4%	0.6301
Hip Fracture Ever	4.8%	5.2%	2.8%	0.0031
Hypertension Ever	45.5%	46.4%	40.6%	0.0021
**Functional Status**				
Bothered by Pain Often	32.7%	32.3%	34.9%	0.1379
Difficulty Picking Up a Dime	8.1%	8.5%	6.1%	0.0205
Difficulty Lifting 10 Pounds	32.7%	34.4%	22.8%	< .0001
Difficulty Pushing/Pulling Large Object	33.8%	35.2%	25.5%	< .0001
Difficulty Climbing Flight of Stairs	27.9%	29.6%	18.5%	< .0001
Difficulty Walking Several Blocks	37.4%	39.2%	26.8%	< .0001
Number of ADLs with Difficulty	0.34(0.84)	0.38(0.88)	0.17(0.56)	< .0001
Number of IADLs with Difficulty	0.40(0.93)	0.45(0.97)	0.14(0.51)	< .0001
Depressive Symptoms - None	37.8%	36.4%	45.9%	< .0001
Depressive Symptoms - One to Two	35.7%	35.4%	37.1%	0.3518
Depressive Symptoms - Three or More	26.5%	28.1%	16.9%	< .0001
TICS 7 Score - Zero to Ten	28.4%	30.2%	17.7%	< .0001
TICS 7 Score - Eleven to Thirteen	31.7%	31.3%	34.7%	0.056
TICS 7 Score - Fourteen to Fifteen	39.9%	38.5%	47.7%	< .0001
**Health Lifestyles**				
Never Smoked	47.9%	47.7%	49.5%	0.3481
Ever Drink Alcohol	46.6%	45.0%	55.7%	< .0001
Obese Weight (BMI ≥ 30)	13.6%	13.9%	12.2%	0.1922
Over Weight (25 ≤ BMI < 30)	36.5%	35.3%	43.3%	< .0001
Normal Weight	46.1%	46.6%	43.2%	0.076
Under Weight (BMI < 18.5)	3.8%	4.2%	1.2%	< .0001
**Self-Rated Health**				
Excellent	10.4%	9.8%	13.5%	0.0017
Very Good	23.3%	21.9%	31.7%	< .0001
Good	30.8%	30.9%	30.2%	0.691
Fair	23.1%	24.0%	18.3%	0.0003
Poor	12.3%	13.4%	6.2%	< .0001
**Utilization of Health Services in Prior 12 months**				
Number of Hospitalizations	22.8%	23.8%	17.1%	< .0001
Number of Physician Visits - One or Less	24.4%	24.8%	22.4%	0.1411
Number of Physician Visits - Two to Three	27.7%	27.3%	29.6%	0.1731
Number of Physician Visits - Four to Six	27.8%	27.6%	28.9%	0.4648
Number of Physician Visits - Seven or More	20.1%	20.3%	19.1%	0.4471
Supply of Physicians per 1000 - County level	2.18(1.74)	2.23(1.8)	1.91(1.36)	< .0001

Note: Standard deviations for continuous variables are in parentheses. The p-values for categorical variables are Chi-Square tests of association and for continuous variables they are two-tailed t-tests. Physicians are defined as MDs.

Of the disease conditions, on average fewer chiropractic users had been told they had a heart condition, diabetes, lung disease, hip fracture, or hypertension. A higher percentage of chiropractic users had zero comorbid conditions while a lower percentage had three or more comorbid conditions. Among functional status measures, chiropractic users had a lower mean value for functional limitations such as difficulty picking up a dime, difficulty lifting ten pounds, difficulty pushing or pulling large objects, difficulty climbing one flight of stairs, and difficulty walking several blocks. Chiropractic users had lower ADL and IADL means, indicating fewer difficulties with activities of daily living and instrumental activities of daily living. In the depressive symptoms categories, chiropractic users on average had fewer depressive symptoms. A higher proportion of chiropractic users relative to nonusers also had higher TICS-7 scores, reflecting higher cognitive functioning. Among the health lifestyle factors a higher percentage of chiropractic users relative to nonusers drank alcohol and were overweight. On average, more chiropractic users rated their health as excellent or very good compared to nonusers, and fewer had any hospitalizations in the prior twelve months. A greater proportion of chiropractic users relative to nonusers also lived in counties with fewer physicians per 1,000 capita

### Binomial Logistic Regression Model

Table 2 shows the adjusted odds ratios (AORs) and 95% confidence intervals for all covariates in the model. Statistically significant effects are shown in bold face.

**Table 2:** Adjusted Odds Ratios for chiropractic use

	AOR (95% CIs)	Reference Category
**Demographic Factors**		
***Age***	***0.981 (0.964 - 0.998)***	
***Male***	***0.759 (0.620 - 0.929)***	***Female***
Race		
***African American***	***0.268 (0.163 - 0.439)***	***White***
Hispanic	0.916 (0.533 - 1.574)	White
***Other***	***0.131 (0.021 - 0.798)***	***White***
Marital Status		
***Never Married***	***0.547 (0.314 - 0.954)***	***Married***
Separated/Divorced	0.806 (0.521 - 1.246)	Married
***Widowed***	***0.758 (0.612 - 0.939)***	***Married***
**Socioeconomic Factors**		
Educational Attainment		
Grade School	1.229 (0.932 - 1.620)	Some High School
High School	1.004 (0.786 - 1.283)	Some High School
Some College	0.904 (0.692 - 1.180)	Some High School
Income Distribution		
Income - First Quintile	0.760 (0.516 - 1.119)	Fifth Quintile
Income - Second Quintile	0.816 (0.627 - 1.060)	Fifth Quintile
Income - Third Quintile	0.769 (0.579 - 1.021)	Fifth Quintile
Income - Fourth Quintile	0.872 (0.695 - 1.095)	Fifth Quintile
One or More Health Insurance Policy	0.849 (0.672 - 1.073)	Zero Additional Health Policies
Working for Pay	0.959 (0.737 - 1.248)	
***Able to Drive a Car***	***1.923 (1.486 - 2.488)***	
**Geographic Location Measures**		
***Northeast***	***0.491 (0.306 - 0.787)***	***MidWest Region***
New York/New Jersey	0.692 (0.472 - 1.013)	MidWest Region
***Mid-Atlantic***	***0.473 (0.307 - 0.727)***	***MidWest Region***
***Southeast***	***0.555 (0.387 - 0.796)***	***MidWest Region***
***North-Central***	***0.721 (0.516 - 0.987)***	***MidWest Region***
***South-West***	***0.369 (0.242 - 0.560)***	***MidWest Region***
***Mountain***	***0.258 (0.140 - 0.478)***	***MidWest Region***
***West***	***0.677 (0.460 - 0.997)***	***MidWest Region***
Pacific-Northwest	0.990 (0.620 - 1.581)	MidWest Region
Rural	1.127 (0.901 - 1.409)	
Distance to Chiropractic College - Far	1.068 (0.878 - 1.298)	Near (< 150 miles)
**Disease History and Comorbidity**		
Zero Comorbid Conditions	0.851 (0.619 - 1.170)	One Comorbid Condition
Two Comorbid Conditions	1.174 (0.859 - 1.603)	One Comorbid Condition
***Three or More Comorbid Conditions***	***1.833 (1.056 - 3.183)***	***One Comorbid Condition***
Arthritis Ever	1.088 (0.808 - 1.466)	
Cancer Ever	0.790 (0.571 - 1.092)	
Heart Condition Ever	0.763 (0.565 - 1.031)	
Diabetes Now	0.856 (0.609 - 1.202)	
***Lung Disease Ever***	***0.607 (0.416 - 0.885)***	
Psych Problems Ever	0.965 (0.662 - 1.407)	
Hip Fracture Ever	0.783 (0.492 - 1.244)	
***Hypertension Ever***	***0.679 (0.506 - 0.912)***	
**Functional Status**		
***Bothered by Pain Often***	***1.541 (1.267 - 1.874)***	
Difficulty Picking Up a Dime	1.015 (0.721 - 1.428)	
Difficulty Lifting 10 Pounds	0.902 (0.706 - 1.152)	
Difficulty Pushing/Pulling Large Object	0.899 (0.713 - 1.135)	
Difficulty Climbing Flight of Stairs	1.086 (0.840 - 1.402)	
Difficulty Walking Several Blocks	0.917 (0.727 - 1.155)	
Number of ADLs with Difficulty	0.882 (0.748 - 1.041)	
***Number of IADLs with Difficulty***	***0.837 (0.705 - 0.993)***	
Depressive Symptoms - One or Two	1.023 (0.851 - 1.229)	No Depressive Symptoms
***Depressive Symptoms - Three or More***	***0.789 (0.612 - 1.016)***	***No Depressive Symptoms***
TICS 7 Score - Zero to Ten	0.887 (0.696 - 1.129)	Eleven to Thirteen TICS 7 Score
TICS 7 Score - Fourteen to Fifteen	0.913 (0.761 - 1.096)	Eleven to Thirteen TICS 7 Score
**Health Lifestyles**		
Never Smoked	1.162 (0.973 - 1.387)	Current or Former Smoker
Ever Drink Alcohol	1.162 (0.976 - 1.385)	Never Drink Alcohol
Obese Weight (BMI ≥ 30)	0.985 (0.757 - 1.281)	Normal Weight
***Over Weight (25 ≤ BMI < 30)***	***1.259 (1.058 - 1.499)***	***Normal Weight***
Under Weight (BMI < 18.5)	0.468 (0.241 - 0.910)	Normal Weight
**Self-Rated Health**		
***Excellent***	***1.613 (1.026 - 2.536)***	***Poor Self-Rated Health***
***Very Good***	***1.658 (1.108 - 2.481)***	***Poor Self-Rated Health***
Good	1.227 (0.839 - 1.797)	Poor Self-Rated Health
Fair	1.066 (0.736 - 1.544)	Poor Self-Rated Health
**Utilization of Health Services in Prior 12 months**		
Number of Hospitalizations	0.837 (0.671 - 1.043)	
Number of Physician Visits - One or Less	0.853 (0.681 - 1.069)	Two to Three Physician Visits
Number of Physician Visits - Four to Six	1.214 (0.981 - 1.502)	Two to Three Physician Visits
***Number of Physician Visits - Seven or More***	***1.415 (1.098 - 1.823)***	***Two to Three Physician Visits***
***Supply of Physicians per 1000 - County level***	***0.916 (0.853 - 0.984)***	

Note: Physician visit counts are in response to the question of how many visits to medical doctors the individual had made. Significant predictors at the 5% level are shown in ***bold face italics***.

The odds of using chiropractic were lower for men, African-Americans, and other non-Hispanic races. For people who never married or were widowed, the odds of chiropractic use were lower relative to those who were married. Respondents with three or more comorbid conditions were 1.83 times more likely than those with one comorbid condition to be a chiropractic user. People with lung disease and hypertension were at lower odds of using chiropractic, as well as those with higher mean IADLs. People who experienced pain often had 1.5 times the odds of using chiropractic relative to those not experiencing pain often. People who were able to drive at baseline had almost two times the odds of those not able to drive of using chiropractic. Among the weight categories, people that were overweight (relative to normal weight) were 1.26 times more likely to be a chiropractic user, while those who were underweight were at lower odds of using chiropractic. People with "very good" and "excellent" self-rated health had 1.6 times the odds of using chiropractic relative to those with "poor" self-rated health. High physician service utilizers, as measured by those with greater than seven visits to a physician in the past year, were 1.4 times more likely than those with two to three visits in the prior year to use chiropractic. Geographically, people living in the Northeast, Midatlantic, Southeast, Southwest, Mountain, and West regions had lower odds of using chiropractic than those living in the Midwest. Respondents living in counties with a higher number of physicians per capita had slightly lower odds (0.916) of using chiropractic. The C-statistic for the model was 0.715, indicating a good fit of the model to the data. The Hosmer and Lemeshow goodness-of-fit test provided additional support based on a p-value of 0.1651, indicating no evidence of a lack of fit to the data.

### Patterns of Chiropractic Use

Forty-two percent of the 806 chiropractic users made their visits over a single calendar year. Nearly 20% of chiropractic users had visits over a two-year period, while 38% had visits spanning three or more calendar years, indicating substantial heterogeneity in the consistency of chiropractic use over time.

**Figure 1:**
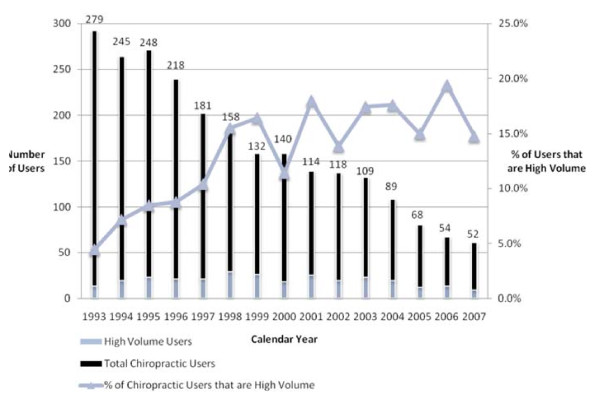
**Distribution of chiropractic users**.

**Figure 2:**
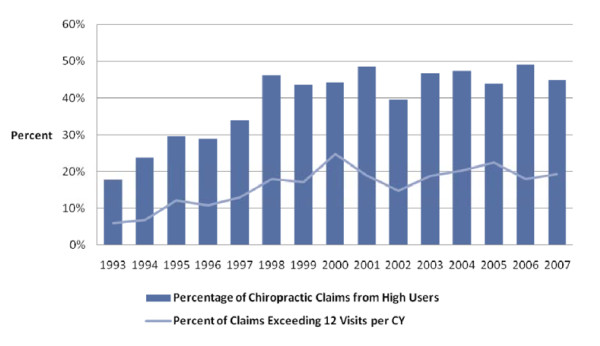
**Percentage of chiropractic claims from high users and percentage exceeding Medicare's "soft cap" of 12 visits per calendar year**.

Of the 806 respondents who used chiropractic, there were 130 users (16%) who exceeded Medicare's "soft cap" of 12 chiropractic visits in any of the calendar years in which they used chiropractic. Although small, this high volume user group accounted for nearly 58% of the total chiropractic claims volume (9,080 out of 15,716). On a calendar year basis the portion of all chiropractic users that exceeded the "soft cap" of 12 chiropractic visits (high volume user group) grew from 4.6% in 1993 to almost 15% by 2007, although this percentage in part reflected a declining number of people in the total chiropractic user group due to death and censoring while the number per year of high volume users was relatively flat over the period. For the same reasons, high volume chiropractic users' claim volume in each calendar year grew from 18% in 1993 to over 45% in 2007, averaging about 45% of the total chiropractic claim volume in the 1998-2007 period. Approximately 15% of the claims from this group would be disallowed if a "hard cap" threshold of 12 visits per calendar year was enforced. Figure 1 shows the distribution of chiropractic users by year and the percentage of high volume users over the 15-year period. Figure 2 shows the annual claim volume attributable to high volume users and the percentage of claims that exceed a 12 visit threshold.

Descriptively, the high volume chiropractic users were on average younger than the lower volume users, had a higher mean proportion of one or more health insurance policies, a lower mean for IADLs, and more often lived in the North-Central region of the United States compared to lower volume users. Logistic regression revealed that high volume users had 0.516 the odds of lower volume users of having a high school education relative to "some high school". High volume users were almost 2.5 times more likely than lower volume users to have arthritis, and about 2.4 times more likely to have difficulty picking up a dime. The C-statistic for the model was 0.707. The Hosmer and Lemeshow test p-value was 0.2550, an indication of adequate fit between model and data. The AORs are reported in Table 3.

**Table 3:** Adjusted Odds Ratios for high volume vs. lower volume chiropractic use

	High Volume Use (> 12 visits per CY)	
	AOR (95% CIs)	Reference Category
**Demographic Factors**		
Age	0.953 (0.905-1.003)	
Male	0.742 (0.426-1.294)	Female
Race		
African American	0.813 (0.164-4.035)	White
Hispanic	0.329 (0.044-2.472)	White
Other	> 999.99 (< 0.001- > 999.99)	White
Marital Status		
Never Married	1.182 (0.263-5.311)	Married
Separated/Divorced	0.700 (0.149-3.298)	Married
Widowed	1.421 (0.791-2.555)	Married
**Socioeconomic Factors**		
Educational Attainment		
Grade School	1.115 (0.551-2.256)	Some High School
***High School***	***0.516 (0.282-0.943)***	***Some High School***
Some College	0.556 (0.290-1.067)	Some High School
Income Distribution		
Income - First Quintile	0.588 (0.185-1.870)	Fifth Quintile
Income - Second Quintile	0.780 (0.386-1.578)	Fifth Quintile
Income - Third Quintile	0.934 (0.452-1.931)	Fifth Quintile
Income - Fourth Quintile	0.789 (0.443-1.405)	Fifth Quintile
One or More Health Insurance Policy	1.752 (0.837-3.665)	Zero Additional Health Policies
Working for Pay	0.978 (0.488-1.958)	
Able to Drive a Car	0.866 (0.416-1.800)	
**Geographic Location Measures**		
Northeast	0.654 (0.157-2.735)	MidWest Region
New York/New Jersey	1.594 (0.562-4.520)	MidWest Region
Mid-Atlantic	1.650 (0.546-4.982)	MidWest Region
Southeast	0.790 (0.296-2.107)	MidWest Region
North-Central	1.903 (0.844-4.290)	MidWest Region
South-West	1.153 (0.402-3.308)	MidWest Region
Mountain	0.539 (0.051-5.698)	MidWest Region
West	1.818 (0.639-5.169)	MidWest Region
Pacific-Northwest	0.651 (0.170-2.491)	MidWest Region
Rural	1.232 (0.691-2.196)	
Distance to Chiropractic College - Far	1.612 (0.953-2.725)	Near (< 150 miles)
**Disease History and Comorbidity**		
Zero Comorbid Conditions	1.613 (0.674-3.863)	One Comorbid Condition
Two Comorbid Conditions	0.568 (0.240-1.346)	One Comorbid Condition
Three or More Comorbid Conditions	0.854 (0.182-4.011)	One Comorbid Condition
***Arthritis Ever***	***2.488 (1.097-5.645)***	
Cancer Ever	1.007 (0.392-2.582)	
Heart Condition Ever	0.906 (0.388-2.117)	
Diabetes Now	0.976 (0.369-2.584)	
Lung Disease Ever	0.790 (0.264-2.366)	
Psych Problems Ever	1.510 (0.532-4.290)	
Hip Fracture Ever	0.613 (0.126-2.975)	
Hypertension Ever	1.202 (0.513-2.818)	
**Functional Status**		
Bothered by Pain Often	0.835 (0.506-1.380)	
***Difficulty Picking Up a Dime***	***2.389 (1.080-5.281)***	
Difficulty Lifting 10 Pounds	0.843 (0.466-1.595)	
Difficulty Pushing/Pulling Large Object	1.321 (0.743-2.347)	
Difficulty Climbing Flight of Stairs	0.987 (0.512-1.902)	
Difficulty Walking Several Blocks	1.057 (0.575-1.944)	
Number of ADLs with Difficulty	0.817 (0.491-1.357)	
Number of IADLs with Difficulty	0.532 (0.255-1.112)	
Depressive Symptoms - One or Two	0.865 (0.529-1.414)	No Depressive Symptoms
Depressive Symptoms - Three or More	1.009 (0.498-2.043)	No Depressive Symptoms
TICS 7 Score - Zero to Ten	0.834 (0.428-1.627)	Eleven to Thirteen TICS 7 Score
TICS 7 Score - Fourteen to Fifteen	1.112 (0.697-1.773)	Eleven to Thirteen TICS 7 Score
**Health Lifestyles**		
Never Smoked	0.802 (0.498-1.292)	Current or Former Smoker
Ever Drink Alcohol	0.860 (0.542-1.363)	Never Drink Alcohol
Obese Weight (BMI ≥ 30)	0.698 (0.340-1.432)	Normal Weight
Over Weight (25 ≤ BMI < 30)	0.888 (0.555-1.421)	Normal Weight
Under Weight (BMI < 18.5)	0.851 (0.131-5.584)	Normal Weight
**Self-Rated Health**		
Excellent	0.932 (0.335-2.597)	Poor Self-Rated Health
Very Good	1.117 (0.442-2.822)	Poor Self-Rated Health
Good	1.240 (0.515-2.986)	Poor Self-Rated Health
Fair	1.131 (0.479-2.672)	Poor Self-Rated Health
**Utilization of Health Services in Prior 12 months**		
Number of Hospitalizations	1.172 (0.726-1.893)	
Number of Physician Visits - One or Less	1.567 (0.987-2.488)	Two to Three Physician Visits
Number of Physician Visits - Four to Six	1.107 (0.711-1.726)	Two to Three Physician Visits
Number of Physician Visits - Seven or More	1.556 (0.918-2.636)	Two to Three Physician Visits
Supply of Physicians per 1000 - County level	1.024 (0.880-1.192)	

Note: Physician visit counts are in response to the question of how many visits to medical doctors the individual had made. Significant predictors at the 5% level are shown in ***bold face italics***.

### Distribution of Specialty Providers

High volume users saw chiropractors more than any other specialty provider covered by Medicare. Among all provider claims for this group, chiropractic ranked first with 21.5% of the total volume, more than internal medicine and family practice combined. Comparatively, chiropractic claims volume was only 4.1% of the total claims volume for the lower volume chiropractic user group, similar to the distribution pattern of the nonuser group. Table 4 shows the distribution of the top most frequently seen provider specialties by user and nonuser groups.

**Table 4:** Specialty provider distribution across chiropractic users and nonusers

*Specialty*	Lower Volume Users (N = 676)	High Volume Users (N = 130)	Non Users (N = 4,704)
Internal Medicine	13.1%	10.6%	15.5%
Family Practice	10.3%	9.8%	9.6%
Clinical Lab	9.0%	6.3%	9.9%
Diagnostic Radiology	8.8%	7.2%	9.5%
Cardiology	7.1%	5.2%	7.4%
Opthalmology	5.6%	4.3%	5.7%
Chiropractic	4.1%	21.5%	0.0%
*Sum of Specialty Percentages Listed*	*58.1%*	*64.9%*	*57.6%*
*Total Claim Volume*	*6,636*	*9,080*	*759,616*

Note: Not all specialty codes are listed so sum of specialties does not add up to 100%.

## Discussion

In this article we examined chiropractic use over a 15-year period using a large nationally representative sample of Medicare beneficiaries in the United States to identify factors associated with chiropractic use and different levels of volume utilization. Our analysis provides several insights of interest. First, we find the average annual prevalence of chiropractic to be 4.8% (range 4.1% to 5.4%) which is lower than previous estimates of national adult chiropractic use, but consistent with Medicare's estimate of use in their covered population [15] and estimates in prior work by Wolinsky and colleagues [9]. We find that those who were less healthy on certain dimensions were more likely to use chiropractic. We also find that those living in specific regions of the country, along with certain measures of access, predict chiropractic utilization. Lastly we find that high volume users, defined as those that exceed the "soft cap" set out by CMS, have distinctly more chiropractic visits than any other provider type, though there are few characteristics that differentiate those who are high volume users from those who are low volume users.

While a higher number of comorbid conditions predicted being a chiropractic user, interestingly there was no difference in objective measures of physical function. Given that reporting being bothered by pain often was predictive of chiropractic use, this may indicate that using chiropractic helps maintain function even in the presence of painful conditions. Self-rated health of very good or excellent was also associated with chiropractic use, suggesting chiropractic users were subjectively healthier, in spite of having a higher number of comorbidities.

With respect to access, those who had more physicians per 1000 individuals in their county were less likely to use chiropractic, which could be due to competition or coordination effects in the market, or that chiropractors serve as alternative source of primary care when physicians are in short supply. We would argue that coordination is the case, given that the bulk of chiropractic claims are for chiropractic manipulation and that those who were the highest users of physicians in the twelve months prior to baseline were more likely to be chiropractic users as well. Those living in rural areas had no higher odds of using chiropractic, in contrast with findings from regional studies of chiropractic use [29,30]. People living outside the Midwest and Pacific Northwest were less likely to use chiropractic, which was consistent with other work on regional patterns of use [30].

Conditional upon any chiropractic use, however, there was not much difference between those who exceeded Medicare's "soft cap" of 12 visits per year and those with fewer annual visits. Indeed, after adjusting for all covariates the only significant differences among chiropractic user types were that high volume users were less likely than lower volume users to have a high school education relative to 'some high school', but were more likely to suffer from arthritis and fine motor function limitations as measured by difficulty picking up a dime. The presence of a painful and chronic health condition like arthritis, along with some limited physical functioning, fit naturally within a model predicting high volume chiropractic use.

The patterns of chiropractic utilization over the 15-year period indicate two distinct groups of users. While 42% of users had visits occurring in just a single calendar year, a second mode of chiropractic utilization occurred where 38% of users sought care over three or more years, reflecting a more persistent use pattern. This latter group warrants further investigation to determine if the patterns are driven by the chronic health needs and geographical access differentials among health care providers (as the evidence would indicate), or whether their utilization is driven by other reasons, such as seeking to "maintain" health. The former reason would be justified for Medicare coverage under current policy, while the latter would not [15].

The high claim volume, the types of procedures performed, and the relative ranking (among all service providers) of chiropractor-provided care in the high volume user group--taken in tandem with this group's higher odds of having arthritis and mobility limitations--paints a picture of a group of older individuals seeking on-going chiropractic care to address chronic and painful health conditions. A separate analysis of procedure code distributions in the two different volume groups provided some clues into the different health needs of the two types of users. The high volume user group had a heavier concentration of two specific procedure codes that relate to manipulation of multiple spinal regions (989.41 and 989.42), indicating complex conditions that potentially justify higher utilization by these subjects. Given that those with more education had a protective effect against being a high volume user, and arthritis increased the odds of being high volume, some of this difference could be potentially tied up in lifetime accumulation of musculoskeletal stress, assuming of course that higher levels of education are correlated with occupations that reduce musculoskeletal stress.

From a policy perspective, if Medicare were to go from their current "soft cap" policy to enacting a "hard cap" on chiropractic utilization based on a threshold of 12 annual visits, the claims volume for chiropractic might drop by 15% per calendar year. If the people most affected by this policy change were to instead substitute other medical providers for their care, (rather than go without care at all), Medicare may see an increase in expenditures on other, better compensated providers. The profile of subjects in the high volume group suggests that their seeking of chiropractic services is driven by both medical need and geographical access limitations to physicians, so enforcing a "hard cap" on this group could have unintended health consequences. Moreover such a policy change would affect a relatively small group of intense older chiropractic users.

## Limitations

There are several limitations to our study. First, AHEAD does not contain measures of health beliefs and preferences for chiropractic. Having such a measure could provide insightful differences between those who seek chiropractic care versus those who do not. Second, we did not have good measures of local chiropractor supply. While the distance to nearest chiropractic college measures chiropractor diffusion and availability, it was not significant in any of the models. This may be because it genuinely doesn't reflect chiropractic supply or familiarity with chiropractic practice (thereby influencing demand), or because it was crudely categorized. A third limitation is that our analyses are based on chiropractic claims volume, not on episodes of care. In future analyses, a distinction should be made between high volume users whose claims reflect an 'episode' of care versus those whose patterns of use indicate regular, periodic chiropractic maintenance treatments. The latter are more exactly the target of Medicare's "soft cap" policy.

## Conclusion

Chiropractic utilization is not monotonic among older adults. Distinct patterns can be discerned between those who use frequently and persistently versus those who use neither frequently nor persistently, with only a handful of characteristics (i.e. more education, having arthritis and fine motor skills limitations) distinguishing high from low volume users. In light of a specific Medicare policy that uniformly covers all eligible beneficiaries, the differences in chiropractic utilization within the AHEAD cohort is an interesting study of utilization variability and the factors that influence that variability. Future research would benefit from data on occupation and other factors that determine lifetime accumulation of musculoskeletal stress.

## Competing interests

The authors declare that they have no competing interests.

## Authors' contributions

PW, JMH and FDW took part in all facets of the study. SEB and MO helped conceptualize the analysis, construct the data and interpret the analysis and critical review of the final manuscript. MPJ helped conceptualize the analysis as well as reviewing the analysis and providing critical review of the interpretation in the manuscript. BK, RLO, GER, and RBW participated in the conceptualization of the study and critical review of the manuscript. All authors have read and approved the manuscript.
